# Label-free quantitative proteomics identifies Smarca4 is involved in vascular calcification

**DOI:** 10.1080/0886022X.2019.1591997

**Published:** 2019-04-11

**Authors:** Chan Wang, Yun Tang, Yanmei Wang, Guisen Li, Li Wang, Yi Li

**Affiliations:** a Department of Nephrology, Sichuan Academy of Medical Sciences and Sichuan Provincial People’s Hospital, School of Medicine, University of Electronic Science and Technology of China, Chengdu, China;; b Institute of Organ Transplantation, Sichuan Academy of Medical Science and Sichuan People’s Hospital, Chengdu, China;; c Department of Nephrology, Affiliated Hospital of North Sichuan Medical College, Nanchong, China

**Keywords:** Proteomics, label-free, Smarca4, vascular calcification

## Abstract

Vascular calcification (VC) is a pathological process characterized by abnormal deposition of calcium phosphate, hydroxyapatite and other mineral substances in the vascular wall. Hyperphosphorus is an important risk factor associated with VC in the general population and patients with chronic kidney disease (CKD). However, there is still a lack of early biomarkers for hyperphosphorus induced VC. We established a calcific rat aorta vascular smooth muscle cells (RASMCs) model by stimulating with β-glycerophosphate. Then we performed label-free quantitative proteomics combined with liquid chromatograph–mass spectrometer/mass spectrometer (LC-2D-MS/MS）analysis and bioinformatics analysis to find the potential biomarkers for VC. In the current study, we identified 113 significantly proteins. Fifty six of these proteins were significantly up-regulated and the other 57 proteins were significantly decreased in calcific RASMCs, compared to that of normal control cells (fold-change (fc)>1.2, *p* < .05). Bioinformatics analysis indicated that these significant proteins mainly involved in the placenta blood vessel development and liver regeneration. Their molecule function was cell adhesion molecule binding. Among them, Smarca4 is significantly up-regulated in calcific RASMCs with fc = 2.72 and *p* = .01. In addition, we also established VC rat model. Real-time quantitative PCR analysis confirmed that the expression of Smarca4 was significantly increased in the aorta of calcified rat. Consistent with the up-regulation of Smarca4, the expression of VC associated microRNA such as miR-133b and miR-155 was also increased. Consequently, our study demonstrates that Smarca4 is involved in hyperphosphorus-induced VC. This finding may contribute to the early diagnosis and prevention of VC.

## Introduction

Vascular calcification (VC) is a common complication associated with chronic kidney disease (CKD), it is also the major cause of cardiovascular disease (CVD) in patients with ESRD [1]. VC is defined as the inappropriate and pathological deposition of mineral in the vascular tissues [2]. As an active cell-mediated pathology process, VC is associated with the process of vascular smooth muscle cells (VSMCs) differentiation into osteoblast-like cells, apoptosis of VSMCs, loss of calcification inhibitors, matrix vesicle release of VSMCs and extracellular matrix degradation [2–4].

In addition, mineral and bone disorders are also closely related to VC in patients with CKD. Renal dysfunction in CKD patients will lead to hyperphosphatemia due to reduced phosphorus clearance [4]. The increase of serum phosphorus may cause secondary disorder of calcium and phosphorus metabolism, then result in an abnormality of mineral and bone metabolism. Hyperphosphatemia also can promote osteoblast-like phenotype transformation and apoptosis of VSMCs, these abnormal VSMCs can release matrix vesicles which is rich in calcium phosphate to further cause VC [5,6]. A meta-analysis revealed that every 1.0 mg/dL of serum phosphorus was associated with 18% increase in the risk of death in patients with CKD [7].

Therefore, control of blood phosphorus is necessary for CKD patients who have VC. At present, the control of hyperphosphatemia mainly includes the following methods: (i) limiting phosphorus absorption in diet, (ii) using phosphorus binder, and (iii) controlling hyperparathyroidism for patients with ESRD. It is also necessary to select appropriate dialysate and ensure adequate dialysis volume [8]. These treatments can improve VC, but the long-term effect has not yet been satisfactory. It is suggested that early diagnosis and prevention of VC should be carried out in CKD patients with high blood phosphorus. Although the risk of VC can predict by testing hydroxyapatite levels, some studies suggest that these biomarkers are not effective due to age and classical risk factors [9]. Therefore, there is still a lack of reliable biomarkers associated with early VC in hyperphosphorus state.

Proteomics is a scientific approach of analyzing and identifying large-scale proteins based on mass spectrometry (MS). Techniques for determining proteomics have chemical labeling methods, such as iTRAQ [10], ICAT [11], and mTRAQ [12]. However, there are some shortcomings of labeling quantification. For example, labeling reagents are expensive, the number of labeled samples is limited and low abundance peptides are difficult to detect. As an alternative method, label-free quantification (LFQ) can avoid such defects. Label-free quantification proteomics usually has a high analytical depth and dynamic range, giving this method an advantage when large global protein changes between different treatments [13]. In order to find novel potential biomarkers for early prevention and diagnosis of hyperphosphorus-induced VC, we used LFQ combined with LC–MS/MS to detect differential proteins between calcific rat aorta vascular smooth muscle cells (RASMCs) and normal RASMCs.

## Materials and methods

### Cell culture

Rat aortic vascular smooth muscle cells were obtained from ScienCell Research Laboratories (ScienCell, San Diego, CA). RASMCs were cultured in 250 mL Dulbecco's modified Eagle's medium (DMEM) containing 10% heat-inactivated fetal bovine serum (FBS), 100 μg/mL streptomycin and 100 units/mL penicillin. Then cells stimulated for 72 h by 770 mg β-glycerophosphate, 11 mg l-ascorbic acid and 10^−8^ M dexamethasone [14]. Cells without β-glycerophosphate were used as control. All cells were incubated at 37 °C in an atmosphere of 5% CO_2_.

### Establishment of vascular calcification animal model

Fourteen male wild-type Wistar rats aged 8–10 weeks, weighing 200–300 g, were randomly divided into two groups (*n* = 7): normal control group and end stage renal disease (ESRD) VC group. The rat model of ESRD VC was successfully established by intragastric administration of 0.75% adenine and 6.25 mg/kg nicotine combined with 3 × 10^6^ unit vitamin D3 intraperitoneal injection. 3 × 10^6^ units of vitamin D3 (cholecalciferol) were intraperitoneally injected for 30 min before nicotine treatment and then gavaged the rats by 6.25 mg/kg nicotine. Nicotine was administered twice in total, with an interval of 9 h. At the same time, rat model of ESRD VC was fed a 0.75% adenine diet, it can cause chronic renal failure in rats. At the 12th week, the rats were anesthetized with chloral hydrate to collect blood samples. After sacrificed rats, the aortic and renal tissue samples were collected.

### Protein sample preparation

Mix RIPA (25 mM Tris–HCl pH 7.6, 150 mM NaCl, 1% NP-40, 1% sodium deoxycholate, 1% SDS) with protease inhibitor cocktail and 1 mM PMSF immediately before use, chill on ice. Then almost 100 mg tissue (1 × 10^7^ cell) in 1.5 mL tube, add 1000 µL RIPA buffer, homogenize or sonicate to dissolve at 4 °C. Centrifuge 15 min at 4 °C with top speed, transfer the supernatant to new tube and keep it on ice. Mix regent A and B with a ratio of 50:1, 160 µL per well and five wells for calibration curve, one well for blank. Add 20 µL sample (dilute 5–10 times) or calibration standard (BSA, five different concentration levels). Shake and incubate at 37 °C for 30 min, read with 562 nm wavelength. Calculate the protein concentration of each sample according to the calibration curve. Take 100 µg protein for each sample and dilute sample to ∼1 mg/mL. Add 4–6 folds volumes of pre-chilled acetone to alkylated protein and shake on ice for 30 min or incubate at –20 °C overnight. Centrifuge at 4 °C with a speed of 10,000×*g*, discard the supernatant carefully without disturbing the pellet. Wash the pellet twice with ∼200 µL 80% chilled acetone. Add 200 µL 1% SDC + 100 mM ABC, mix with vortex and spin down. Sonicate 5–30 min in water bath to dissolve protein pellet. Add 5 mM TCEP to each sample, incubate and mix at 55 °C for 10 min. Add 10 mM IAA after samples cooling down to RT, incubate in the dark for 15 min. Re-suspend trypsin with re-suspension buffer to 0.5 µg/µL and incubate at RT for 5 min. Add trypsin solution to each sample (protein:trypsin = 50:1). Mix well and spin down, incubate at 37 °C with thermomixer for about 8 h or overnight. Precipitate SDC with 2% TFA (final). Centrifuge at top speed, transfer supernatant to new tube. Add *n* × 100 µL 2% TFA to the pellet, mix to extract co-precipitated peptides. Repeat the washing step twice. Merge 3 supernatants, centrifuge at top speed for 10–20 min, transfer supernatant to new tube carefully. Equilibrate C_18_ column with 500 µL ACN. Wash out ACN with 500 µL 0.1% FA two times, discard the wash out. Load peptide solution to C 18 column, let the solution flow through the column slowly, and collect flow through (A). Repeat the peptide loading step once. Wash column with 1000 µL 0.1% FA, discard the wash out. Elute peptide with 400 µL 70% ACN, collect elution (B) with new tube. Repeat the desalting step once more with flow through (A). Merge 2 elution (B), vacuum dry the elution under 4 °C or RT. The reagents used in this experiment were as follows: TCEP (tris(2-carboxyethyl) phosphine), IAA (iodoacetamide), SDC (sodium deoxycholate), Tris–HCl, C_18_ columns (3M), FA (formic acid, LC–MS), TFA (trifluoroacetic acid, HPLC), PMSF (phenylmethylsulfonyl fluoride), and ABC (ammonium bicarbonate) were from Sigma-Aldrich (St. Louis, MO). ACN (acetonitrile, LC–MS) and H_2_O (LC–MS) were from J.T.Baker (Center Valley, PA). Trypsin (sequence grade) was from Promega (Madison, WI). NP-40 (Nonidet P 40), NaCl, SDS, and acetone were from Sangon Biotech (Shanghai, China). Protease inhibitor cocktail was from Kangchen Bio-tech (Shanghai, China).

### LC–MS/MS analysis

For each sample, ∼2 µg peptides were separated and analyzed with a nano-UPLC (EASY-nLC1200) coupled to Q-Exactive mass spectrometry (Thermo Finnigan, ‎Waltham, MA). Separation was performed using a reversed-phase column (100 µm, ID × 15 cm, Reprosil-Pur 120C 18-AQ, 1.9 µm, Dr. Math). Mobile phases were H_2_O with 0.1% FA, 2% ACN (phase A) and 80% ACN, 0.1% FA (phase B). Separation of sample was executed with a 120 min gradient at 300 nL/min flow rate. Gradient B: 8–30% for 92 min, 30–40% for 20 min, 40–100% for 2 min, 100% for 2 min, 100–2% for 2 min and 2% for 2 min.

Data dependent acquisition was performed in profile and positive mode with Orbitrap analyzer at a resolution of 70,000 (@200 m/z) and *m*/*z* range of 350–1600 for MS1; for MS2, the resolution was set to 17,500 with a dynamic first mass. The automatic gain control (AGC) target for MS1 was set to 3.0E + 6 with max IT 50 ms, and 5.0E + 4 for MS2 with max IT 100 ms. The top 20 most intense ions were fragmented by HCD.

### MaxQuant database search

Raw MS files were processed with MaxQuant (Version 1.5.6.0). The protein sequence database (Uniprot_organism_2016_09) was downloaded from UNIPROT. This database and its reverse decoy were then searched against by MaxQuant software. The quantification type was LFQ with match between run and iBAQ; Trypsin was set as specific enzyme with up to three miss cleavage; oxidation [M] and acetyl [protein N-term] were considered as variable modification (max number of modifications per peptide is 3), carbamidomethyl [C] was set as fixed modification; Both peptide and protein FDR should be less than 0.01. Only unique and razor peptides were used for quantification. All the other parameters were reserved as default.

### Bioinformatics analysis

Proteins significant in this two comparing groups are used to perform clustering analysis. The volcano plot combines a measure of statistical significance from a statistical test with the magnitude of the change, enabling quick visual identification of those data-points. The gene ontology (GO) functional was performed for the differential expression of proteins. Protein–protein interactions (PPIs) were evaluated by the Search Tool for the Retrieval of Interacting Genes (STRING) database.

### Real-time quantitative PCR

We used two-step RT-PCR to identify the differential expression of Smarca4, miR-133b, miR-155, and calcific related gene SM22α, FGF23, OPN in normal rat aortic tissue and calcific rat aortic tissue. After sacrificed rats, the aortic and renal tissue samples were collected and total RNA was extracted by Trizol method. According to the manufacturer’s instruction of TB Green qPCR, the final volume of RT reaction solution is up to 20 µL. Then, reverse transcription of RT reaction solution was carried out under the following conditions: 37 °C, 15 min; 85 °C, 5 s; 4 °C. This reaction was completed through GeneAmp PCR System 9700. We used CFX96 Real-Time PCR Detection System to prepare PCR reaction solution. Added 5 µL cDNA to the PCR reaction solution to make the final volume up to 25 µL, cDNA had been diluted six times before added. The conditions of two-step PCR were as follows: one cycle of 30 s at 95.0 °C followed by 40 cycles of 5 s at 95.0 °C and 30 s at 60.0 °C. The primer sequence of real-time PCR is shown below: Smarca4 (BRG1)-F, GAAGCAGTGGCTCAAGGCTA and Smarca4 (BRG1)-R, GAGGTTAGGTGGGTTTGGGG; miR-155-F, ATTGTGATAGGGGTTTTGGCCT and miR-155-R, TTAATGCTAACAGGTAGGAGTCAGT; miR-133b-F, TGGCTGGTCAAACGGAACCAA and miR-133b-R, GCCCTGCTGTAGCTGGTTGA; SM22α-F, TGGCGTGATTCTGAGCAAGT and SM22α-R, GTTCTTGCTCACGGCCAAAC; FGF23-F, CACTACCTGGTGAGCTTGGG and FGF23-R, CTTCCTCTGCACTCGGTAGC; OPN-F, AGGCACTGAAAGCCGGTGTG and OPN-R, TCAGCAATGACCCAGTTGGC. The primer of BRG1, miR-133b, and miR-155 was obtained from TSLINGKE Biological Technology (Chengdu, China). The primer of SM22-α, FGF23, and OPN was obtained from Sangon Biotech (Shanghai) Co., Ltd. (Shanghai, China).

### Statistical analysis

The SPSS (SPSS for Windows, version 13.0, SPSS Inc., Chicago, IL) was used for statistical analysis. Data were statistically analyzed by one-way ANOVA. Differences were expressed as means ± SD. *p < .05 and #*p* < .01 were regarded as indicating a significant difference.

## Results

### Identification of 113 significant proteins by LFQ analysis

There are 113 significant proteins identified from two groups (Table 1). In calcific RASMCs group, 56 proteins were up-regulated and 57 proteins were down-regulated. Of these, 57 differentially expressed proteins were found according to the 1.2-fold changes (*p* < .05).

**Table 1.** One hundred and thirteen significant proteins were identified between control and calcification group by LFQ analysis.

Majority protein IDs	Gene names	LFQ intensity	Fold-change	*p* Value
A0A0G2K4X8	*Skp1*	32 692 000	1.209496	.037314662
P97608	*Oplah*	7 007 000	1.239621	.003830313
Q920L2	*Sdha*	129 130 000	1.255603	.03068369
F1M6W2	*Ermp1*	88 267 000	1.258816	.039785832
A0A0G2K059	*Mcu*	26 914 000	1.259262	.048846787
Q8R490	*Cdh13*	54 905 000	1.259552	.008855025
G3V8U8	*Bcat2*	37 211 000	1.264705	.042710049
A1L1J9	*Lmf2*	23 403 000	1.271239	.021210873
Q5I0D1	*Glod4*	18 369 000	1.280626	.009431631
Q6WN19	*Rtn2*	33 353 000	1.283646	.046210873
D3ZPW7	*Gpx8*	73 602 000	1.300624	.01276771
Q6AY55	*Dcakd*	23 797 000	1.300956	.016721582
Q6AYK1	*Rnps1*	12 528 000	1.306912	.038962109
A0A0G2JVC8	*Lss*	18 791 000	1.308554	.008113674
Q642E6	*Tpp1*	36 871 000	1.321535	.047116969
Q63083	*Nucb1*	85 541 000	1.325339	.006919275
Q5XIC0	*Eci2*	10 277 000	1.347182	.032125206
Q63524	*Tmed2*	35 129 000	1.358797	.033113674
B5DEF3	*Ggcx*	16 134 000	1.372343	.038509061
Q5EAJ6	*Ikbip*	117 160 000	1.40622	.007331137
Q5U367	*Plod3*	90 475 000	1.409278	.047981878
Q62703	*Rcn2*	28 757 000	1.426199	.040156507
D4A3V2	*Ndufa6*	7 482 200	1.449301	.00815486
M0R6K0	*Lamb2*	18 512 000	1.461328	.034472817
Q66HT5	*Cyr61*	2 150 700	1.564511	.034143328
Q3B7U9	*Fkbp8*	11 299 000	1.572985	.013179572
M0R6N2	*Marc2*	5 026 200	1.578071	.018492586
D3ZY47	*RGD1559896*	0	1.600831	.029283361
F1M1U0	*Mxra7*	14 163 000	1.649825	.001688633
D3ZZ68	*Synpo2l*	15 166 000	1.759235	.048228995
Q642E5	*Mvd*	7 700 900	1.77193	.011779242
Q66HG6	*Ca5b*	3 511 200	1.787539	.031136738
D3Z9E1	*Emilin1*	31 645 000	1.812562	.009596376
A2VD12	*Pbxip1*	19 105 000	1.926187	.010420099
O08628	*Pcolce*	36 407 000	1.946271	.003336079
B1WC25	*Tra2a*	5 398 300	2.009066	.007578254
P14141	*Ca3*	36 616 000	2.029082	.020634267
B7TXW4	*LOC689757*	0	2.047413	.002553542
Q5FVN0	*Lpcat3*	6 372 700	2.068401	.011943987
O09018	*Nr2f2; Nr2f1*	5 797 300	2.098922	.030230643
G3V7V4	*Enpp1*	3 167 900	2.128268	.031260297
Q6AYQ4	*Tmem109*	0	2.170313	.032166392
G3V6Z3	*Afap1*	0	2.173172	.037932455
A0A0G2JW51	*Dhx30*	4 196 100	2.204185	.01684514
Q499R2	*Utp14a*	0	2.438749	.003047776
Q6PEC1	*Tbca*	0	2.485802	.034102142
G3V790	*Smarca4*	0	2.720274	.01169687
Q5PPL3	*Nsdhl*	8 706 700	2.861168	.019975288
P02454	*Col1a1*	160 530 000	2.979068	.041474465
B2RZ27	*Sh3bgrl3*	0	2.997381	.003377265
F1LWQ2	*Fas*	0	3.037187	.005313015
Q4KM64	*Jagn1*	0	3.829429	.043410214
F1MAA1	*Usp47*	18 976 000	4.580024	.012850082
Q6AYS3	*Ctsa*	48 229 000	1.27612	.034019769
F1LTJ5	*Hspg2*	167 060 000	1.363026	.038426689
A0A0G2K678	*Ylpm1*	0	1.578266	.020799012
A0A0G2K0D3		1,299,600	1.640035	.04130972

The table shows majority protein IDs, gene name, LFQ intensity, fold-change, and *p* value. Due to insufficient unique peptide, these proteins cannot be distinguished, so treated as a group, they are sorted by number of identified peptides in descending order. The ‘Majority’ means those proteins that have at least half of the peptides that the leading protein has. Label-free quantification (LFQ) results, corresponds to summed XIC of each sample; normalized by max LFQ algorithm, a global optimization procedure based on achieving the least overall proteome variation.

### Significant proteins under hyperphosphorus-induced calcification

**Figure 1.**
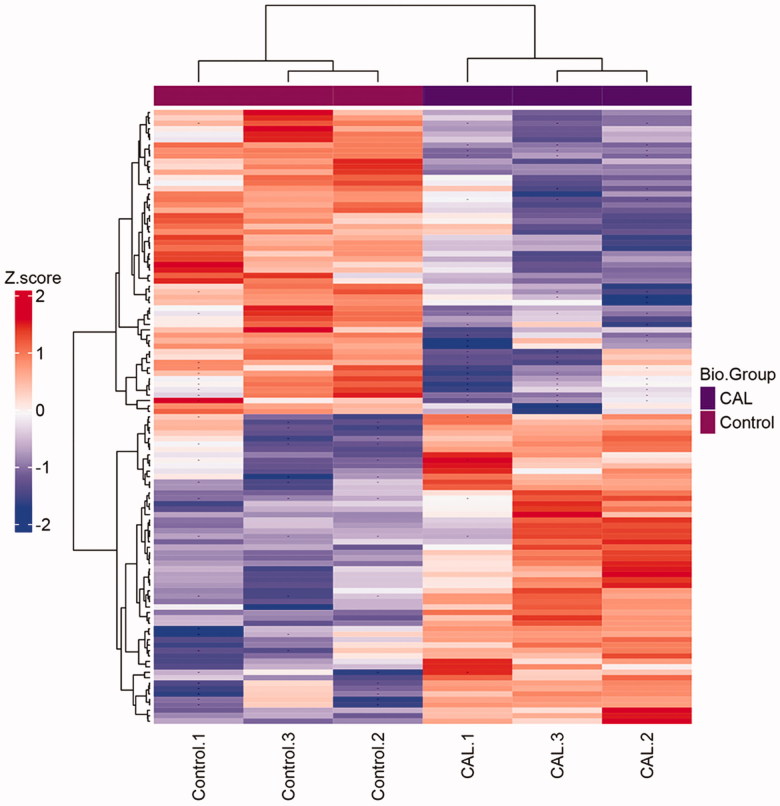
Clustering heatmap of the significant proteins in comparison of CAL – control. If the number of proteins to be shown exceeds a specific value, no protein names would be drawn. Missed values are indicated with '–'. The group of control and CAL has three repeats, respectively.

A total of 113 significant proteins were grouped in clusters analysis between normal RASMCs and calcific RASMCs. After hyperphosphorus-induced calcification, the low expression levels of 56 proteins under normal RASMCs were increased, whereas that of 57 proteins with high expression levels before were decreased (Figure 1). The results showed that the expression of proteins changed significantly after being induced by high phosphorus environment. We used this treatment to induce calcification of RASMCs successfully *in vitro*.

### Changes of significant proteins by volcano blot

**Figure 2.**
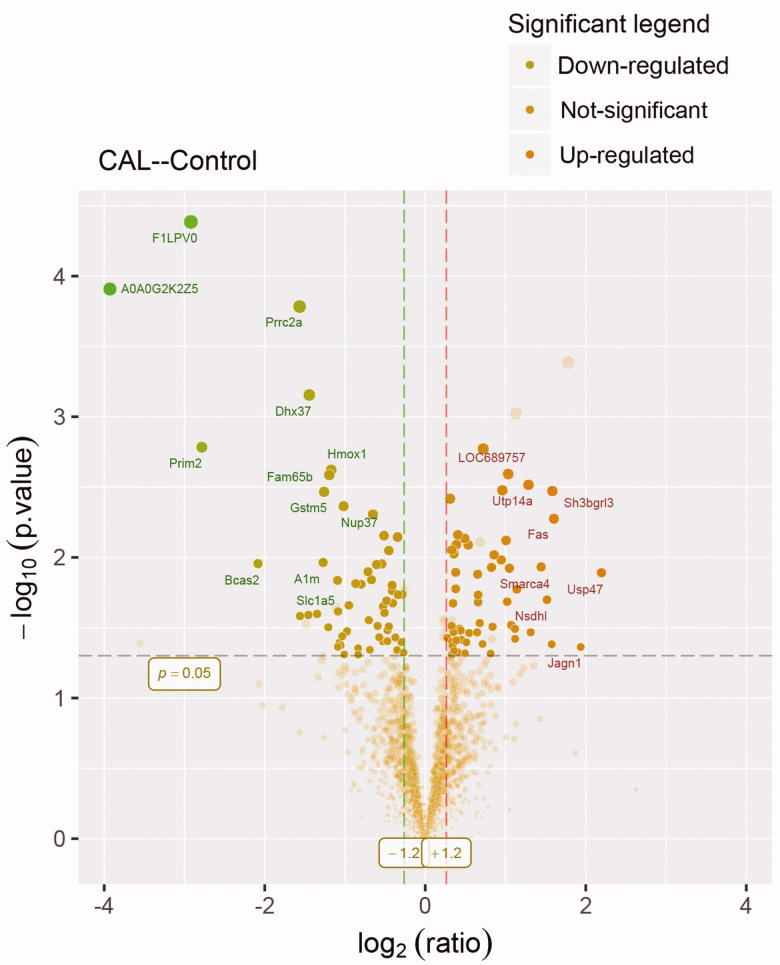
Volcano plot showing proteomics data. These points indicates different proteins that display both large magnitude fold-changes (*x* axis) and high statistical significance ( -log10 of *p* values, *y* axis). Dashed horizontal line shows the *p* values cutoff, and the two vertical dashed lines indicate down/up regulated proteins. Transparent points in the significant region mean these proteins do not satisfy some other conditions.

There are 56 proteins up-regulated by fold-change (fc)>1.2 (*p* < .05) and 57 proteins down-regulated by fc<–1.2 (*p* < .05) (Figure 2). The result is a direct indication of changed significant proteins. A dot is a protein. The fc of up-regulated proteins was around 2.0. We are interested in Smarca4. It is increased by fc = 2.72 (*p* = .01).

### GO analysis of significant proteins

**Figure 3.**
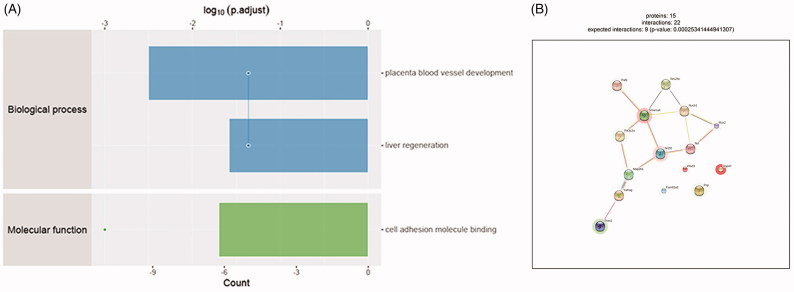
Bioinformatics results analysis. (A) Enriched GO items. Top axis is log10 (adjust *p* values), bottom axis is gene count. The ontology covers two domains. Molecular function: the elemental activities of these significant proteins at the molecular level are cell adhesion molecule binding. Biological process: these differentially expressed proteins are mainly involved in the placenta blood vessel development and liver regeneration. (B) STRINGdb protein-protein network enrichment analysis. The protein-protein interaction network of significant proteins is shown. The interactions include direct (physical) and indirect (functional) associations; they stem from computational prediction, from knowledge transfer between organisms, and from interactions aggregated from other (primary) databases.

To get more insight on the biological significance of these significant proteins in calcific RASMCs, GO analysis was conducted on 113 significant proteins. Biological process analysis shows these significant proteins participate the placenta blood vessel development and liver regeneration. Molecule function is cell adhesion molecule binding (Figure 3(A)). These proteins are involved in the development of placenta blood vessel, may also associate with the pathological process of VC after they increased in adult.

### STRINGdb protein–protein interaction analysis

We used STRINGdb database to search for the identified proteins above thus to observe the interactions between proteins expressed with a significant difference. The red background shows up-regulated and green background shows down-regulated. The depth of colors indicates the degree of change (Figure 3(B)). Smarca4 with red background and interacts with 5 proteins directly. It can promote venous specification by remodeling NR2F2 promoter in embryonic vascular development. This would be a potential regulatory mechanism in VC.

### Establishment of ESRD vascular calcification rat model

**Figure 4.**
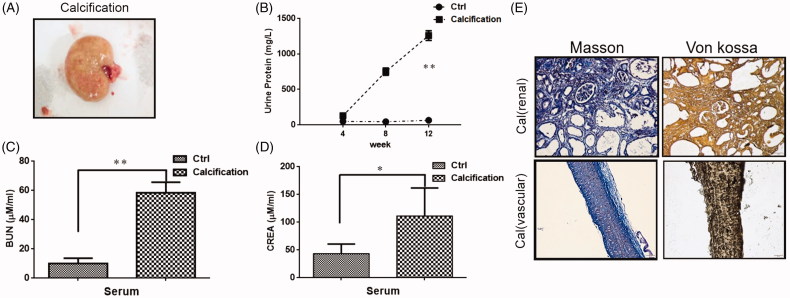
Renal pathological parameters in calcified rats. (A) Renal phenotype of calcified rats. It showed renal hypertrophy, pale color and uneven surface. (B) Comparison of urine protein between calcified rats and normal rats. The urine protein of the calcified group was significantly higher than that in the normal group. With the extension of modeling time, the change is more obvious. (C) Comparison of serum urea nitrogen between calcified rats and normal rats. The serum urea nitrogen concentration in the calcified group was significantly higher than that in the normal group, and the difference was statistically significant. (D) Comparison of serum creatinine between calcified rats and normal rats. The serum creatinine concentration in the calcified group was significantly higher than that in the normal group, and the difference was statistically significant. (E) The calcium deposition of renal and vascular. Masson staining showed a large amount of deposits in renal interstitial tissue calcified aorta (it indicates collagen hyperplasia). The von Kossa staining showed a large amount of black particle deposits in the renal interstitial tissue and calcified aorta (black deposits indicate calcium deposition).

ESRD VC rats showed renal hypertrophy, pale color and uneven surface (Figure 4(A)). The urine protein, BUN and serum creatinine of the VC rats were significantly increased compared with the normal rats (Figure 4(B–D)). At last, Masson staining and von Kossa staining were conducted to evaluate renal calcification and VC. Calcium deposition in calcified rat is significant in both vascular and renal (Figure 4(E)). Therefore, these results indicated that the ESRD VC rat model was successfully constructed.

### Real-time PCR analysis

**Figure 5.**
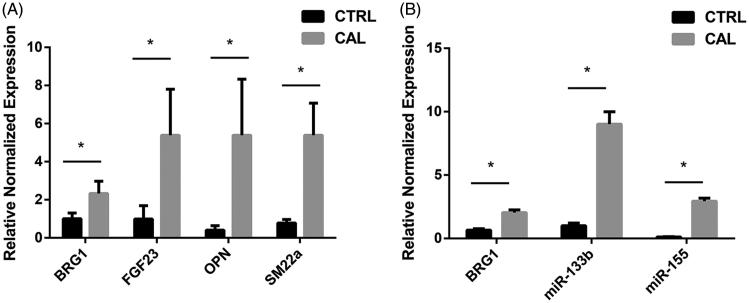
(A) Real-time PCR analysis. The relative normalized expression of Smarca4, miR-155 and miR-133b between normal control group and VC group. (B) The relative normalized expression of Smarca4, FGF23, OPN and SM22α between normal control group and VC group.

We analyzed the changes in the levels of mRNA expression of Smarca4, miR-155, miR-133b, and calcific related gene SM22α, FGF23, and OPN in rat aortic tissue by real-time PCR. The results showed the expression of SM22α, FGF23, and OPN mRNA increased in the calcific aortic tissue, compared with it in the normal aortic tissue (Figure 5(A)). And the mRNA expression of Smarca4, miR-155, and miR-133b showed the same changes, all of them are increased in calcific aortic tissue (Figure 5(B)).

## Discussion

In this study, 113 significant proteins were identified between calcific and control RASMCs by label-free quantitative proteomics. Results of cluster analysis showed the differential proteins. There were three samples in the normal RASMCs and calcific RASMCs respectively, which ensured the reliability of the experimental results. In the normal RASMCs, the protein expression in the three samples was the same before and after β-glycerophosphate treatment, as well as the calcific RASMCs. Among them, 56 proteins were up-regulated and 57 were down-regulated after β-glycerophosphate induced calcification.

Of these, a total of 57 differentially expressed proteins were identified according to the fc > 1.2 (*p* < .05). In volcano plot, the proteins of fc > 1.2 were up-regulated, the proteins of fc<–1.2 were down-regulated, and the proteins below the line of *p* = .05 were excluded. The fc value of the up-regulated proteins was around 2.0, only some of these proteins were marked in the volcano figure.

Bioinformatics analysis showed that these significant proteins were mainly involved in placenta blood vessel development and cell adhesion molecule binding. Curtis et al. identified that conditional deletion of Smarca4 had revealed its importance role in the embryonic vascular development [15]. They further revealed that in the course of embryonic vascular development, Smarca4 promoted venous specification by remodeling the COUP-TFII (chicken ovalbumin upstream promoter transcription factor 2, also known as NR2F2) promoter [16]. In addition, Smarca4 can also bind to cell adhesion molecules. For example, it interacted with and was recruited to the CAM promoters, its over-expression also promoted transactivation of adhesion molecules and leukocyte adhesion induced by inflammatory signals [17]. Based on the results of bioinformatics analysis, we chose Smarca4 for further validation.

Smarca4, also known as BRG1, is the core catalytic subunit of the SWI/SNF. It regulates the transcriptional activity of many genes by altering the chromatin structure, then regulates cell cycle, proliferation and differentiation [18]. Smarca4 can participate in VC by regulating proliferation, apoptosis and phenotypic transformation of VSMCs. Studies have found that the expression of Smarca4 in the aortic middle layer of patients with thoracic aortic aneurysm (TAA) is significantly increased, and in human aortic VSMCs cultured *in vitro*, overexpression of Smarca4 can promote apoptosis and inhibit proliferation of VSMCs [19]. Apoptotic VSMCs can release matrix vesicles, under CKD, these matrix vesicles form a micro-environment of alkaline calcium-phosphorus deposition, increasing hydroxyapatite deposition, and initiating VC. Therefore, the apoptosis of VSMCs induced by Smarca4 may be associated with VC. Furthermore, phenotypic conversion of VSMCs plays a very important role in VC. A research has found that troponin binds to the ATPase subunit of Smarca4 directly and activates VSMCs differentiation-associated genes during VSMCs differentiation [20]. These studies suggest that Smarca4 may be involved in VC, but the specific mechanisms have not been revealed. Thus, there are still many problems waiting to be explored.

At the same time, we found that Smarca4 can affect VC through miRNA. In a model of mice harboring smooth muscle-specific deletion of Smarca4, miR-133b is down-regulated [21]. In addition, the study of Cuadros et al. showed that the expression level of Smarca4 could negatively correlate with miR-155, at least in Burkitt’s lymphoma and diffuse large B cell lymphoma (DLBCL) cell lines [22]. miR-155 is also expressed in activated B and T cells, monocytes, macrophages and ECs and SMCs [23]. And there are many evidence to demonstrate that microRNA plays an important role in hyperphosphorus-induced VC. In a uremia rat model fed with high phosphorus diet, the levels of miR-133b decreased. *In vitro*, similar expression can be found in hyperphosphorus cultured VSMCs [24]. Similarly, in animal model and cultured VSMC from CKD rats, the expression of miR-155 is decreased and negatively correlated with the quantity of calcification in the aorta [25]. According to these studies, we want to understand if there are some connections between Smarca4, miR-133b, and miR-155.

To validate the proteomics results and the relationship between Smarca4, miR-133b, and miR-155, we established ESRD VC rat model. There are three methods commonly used to establish ESRD VC animal models. For example, 5/6 nephrectomy rat model [26], vitamin D3 combined nicotine (VDN) [27], and adenine feeding rats [28]. We have improved our experimental approach on this basis. We used adenine combined with VDN to make rats exhibit ESRD VC while shortening the experiment time. High doses of vitamin D3 can increase the calcium content of arterial tissue, nicotine can enhance the role of vitamin D3. And a large number of adenines form 2,8-dihydroxyadenine under the action of xanthine oxidase, which is extremely insoluble in water. It eventually blocks the renal tubules and cause renal failure. Although this method can induce ESRD VC rat model, it is not suitable for the study of VC induced by calcium and phosphorus metabolism disorder in human ESRD. Our team is also exploring a more suitable ESRD VC animal models.

In order to prove that we successfully constructed a VC rat model *in vivo*, it is necessary to verify the expression of calcification-related indicators SM22α, FGF23, and OPN. RT-PCR analysis showed that the mRNA levels of above three indicators are increased in calcific aortic tissue. FGF23 is a phosphate hormone that regulates the homeostasis of phosphorus *in vivo*, it increases when the kidney function in CKD patients is not sufficient to compensate for high blood phosphorus [29,30]. OPN is an osteochondral gene, it increases when VSMCs change contractile phenotype to osteoblast phenotype. RT-PCR analysis of increased expression of FGF23 and OPN in calcific aortic tissue is the same as other studies [31,32]. While SM22α is a contractile phenotype marker of VSMCs, it should be down-regulated in calcific aortic tissue but our results showed opposite change.

We further amplify RT-PCR to determine whether the expression of Smarca4, miR-133b and miR-155 is changed in calcific aortic tissue. The results showed that the expression of Smarca4 was up-regulated in calcific aortic tissue, which was consistent with the LFQ proteomics analysis. However, the expression of miR-133b and miR-155 was also increased in calcified aortic tissue, which was inconsistent with previous reports [24,25]. But we can find that the variation of Smarca4, miR-133b, and miR-155 was consistent, indicating they may be synergistic in VC.

In conclusion, label-free quantitative proteomics identifies Smarca4 can be novel potential biomarkers in hyperphosphorus-induced VC. Smarca4 is mainly involved in placenta blood vessel development and cell adhesion molecule binding. It plays an important role in early vascular growth and differentiation. In adult, the abnormal expression of Smarca4 may increase the risk of VC in CKD patients with hyperphosphatemia. The pathology process maybe associated with microRNA. But the underline mechanisms of how Smarca4 interacts with microRNA like miR-133b and miR-155 are still unknown. Our study had some defects, we did not verify the interaction between Smarca4 and other proteins shown in STRINGdb. RT-PCR also only quantitatively analyzed the changes of Smarca4, miR-133b, and miR-155, did not reveal the mechanism between them. Thus, the results lack of further analysis of Smarca4 in calcific aortic tissue. More research is needed to support that Smarca4 regulates VC.

Consequently, our study provides a new insight into biomarkers of early diagnosis and prevention of hyperphosphorus-induced VC. In addition, the interaction of Smarca4 with microRNA maybe a potential target for VC.

## References

[1] Karohl C, D'Marco Gascon L, Raggi P Noninvasive imaging for assessment of calcification in chronic kidney disease. Nat Rev Nephrol. 2011;7:567–577. 21862991 10.1038/nrneph.2011.110

[2] Tatsumoto N, Yamada S, Tokumoto M, et al. Spironolactone ameliorates arterial medial calcification in uremic rats: the role of mineralocorticoid receptor signaling in vascular calcification. Am J Physiol Renal Physiol. 2015;309:F967–F979. 26336165 10.1152/ajprenal.00669.2014

[3] Cianciolo G, Capelli I, Cappuccilli M, et al. Calcifying circulating cells: an uncharted area in the setting of vascular calcification in CKD patients. Clin Kidney J. 2016;9:280–286. 26985381 10.1093/ckj/sfv145 PMC4792620

[4] Paloian NJ, Giachelli CM A current understanding of vascular calcification in CKD. Am J Physiol Renal Physiol. 2014;307:F891–F900. 25143458 10.1152/ajprenal.00163.2014 PMC4200295

[5] Libby P, Ridker PM, Hansson GK Progress and challenges in translating the biology of atherosclerosis. Nature. 2011;473:317–325. 21593864 10.1038/nature10146

[6] Gross P, Six I, Kamel S, et al. Vascular toxicity of phosphate in chronic kidney disease: beyond vascular calcification. Circ J. 2014;78:2339–2346. 25077548 10.1253/circj.cj-14-0735

[7] Palmer SC, Hayen A, Macaskill P, et al. Serum levels of phosphorus, parathyroid hormone, and calcium and risks of death and cardiovascular disease in individuals with chronic kidney disease: a systematic review and meta-analysis. JAMA. 2011;305:1119–1127. 21406649 10.1001/jama.2011.308

[8] Cannata-Andia JB, Martin KJ The challenge of controlling phosphorus in chronic kidney disease. Nephrol Dial Transplant. 2016;31:541–547. 25770169 10.1093/ndt/gfv055

[9] Sophie L, Hirokazu O, Lucie D, et al. Vascular calcification in chronic kidney disease: are biomarkers useful for probing the pathobiology and the health risks of this process in the clinical scenario? Nephrol Dial Transplant. 2014;29:1275. 24009287 10.1093/ndt/gft368

[10] Ross PL, Huang YN, Marchese JN, et al. Multiplexed protein quantitation in *Saccharomyces cerevisiae* using amine-reactive isobaric tagging reagents. Mol Cell Proteomics. 2004;3:1154–1169. 15385600 10.1074/mcp.M400129-MCP200

[11] Gygi SP, Rist B, Gerber SA, et al. Quantitative analysis of complex protein mixtures using isotope-coded affinity tags. Nat Biotechnol. 1999;17:994–999. 10504701 10.1038/13690

[12] Un-Beom K, Jeonghun Y, Hoguen K, et al. Quantitative analysis of mTRAQ-labeled proteome using full MS scans. J Proteome Res. 2010;9:3750–3758. 20465265 10.1021/pr9011014

[13] Schulze WX, Usadel B Quantitation in mass-spectrometry-based proteomics. Annu Rev Plant Biol. 2010;61:491–516. 20192741 10.1146/annurev-arplant-042809-112132

[14] Zhang W, Li Y, Ding H, et al. Hydrogen peroxide prevents vascular calcification induced ROS production by regulating Nrf-2 pathway. Ren Fail. 2016;38:1099–1106. 27300444 10.1080/0886022X.2016.1194143

[15] Curtis CD, Davis RB, Ingram KG, et al. Chromatin-remodeling complex specificity and embryonic vascular development. Cell Mol Life Sci. 2012;69:3921–3931. 22618247 10.1007/s00018-012-1023-4 PMC3661716

[16] Davis RB, Curtis CD, Griffin CT BRG1 promotes COUP-TFII expression and venous specification during embryonic vascular development. Development. 2013;140:1272–1281. 23406903 10.1242/dev.087379 PMC3585661

[17] Fei F, Dewei C, Liming Y, et al. Proinflammatory stimuli engage Brahma related gene 1 and Brahma in endothelial injury. Circ Res. 2013;113:986–996. 23963727 10.1161/CIRCRESAHA.113.301296 PMC4049295

[18] Li L, Liu D, Bu D, et al. Brg1-dependent epigenetic control of vascular smooth muscle cell proliferation by hydrogen sulfide. Biochim Biophys Acta (BBA)-Mol Cell Res. 2013;1833:1347–1355. 10.1016/j.bbamcr.2013.03.002 23499876

[19] Shuwei W, Xiwu Z, Yang Y, et al. BRG1 expression is increased in thoracic aortic aneurysms and regulates proliferation and apoptosis of vascular smooth muscle cells through the long non-coding RNA HIF1A-AS1 in vitro. Eur J Cardio-Thorac Surg. 2015;47:439. 10.1093/ejcts/ezu215 24875884

[20] Zhou J, Zhang M, Fang H, et al. The SWI/SNF chromatin remodeling complex regulates myocardin-induced smooth muscle-specific gene expression. ATVB. 2009;29:921. 10.1161/ATVBAHA.109.187229 PMC2730881 19342595

[21] Chen M, Herring BP Regulation of microRNAs by Brahma-related gene 1 (Brg1) in smooth muscle cells. J Biol Chem. 2013;288:6397–6408. 23339192 10.1074/jbc.M112.409474 PMC3585074

[22] Cuadros M, Sanchez-Martin V, Herrera A, et al. BRG1 regulation by miR-155 in human leukemia and lymphoma cell lines. Clin Transl Oncol. 2017;19:1010–1017. 28251496 10.1007/s12094-017-1633-2

[23] Metzinger-Le Meuth V, Burtey S, Maitrias P, et al. microRNAs in the pathophysiology of CKD-MBD: biomarkers and innovative drugs. Biochim Biophys Acta Mol Basis Dis. 2017;1863:337–345. 27806914 10.1016/j.bbadis.2016.10.027

[24] Panizo S, Naves-Diaz M, Carrillo-Lopez N, et al. MicroRNAs 29b, 133b, and 211 regulate vascular smooth muscle calcification mediated by high phosphorus. J Am Soc Nephrol. 2016;27:824–834. 26187577 10.1681/ASN.2014050520 PMC4769184

[25] Chen NX, Kiattisunthorn K, O'Neill KD, et al. Decreased microRNA is involved in the vascular remodeling abnormalities in chronic kidney disease (CKD). PLoS One. 2013;8:e64558. 23717629 10.1371/journal.pone.0064558 PMC3661525

[26] Gretz N, Meisinger E, Strauch M Partial nephrectomy and chronic renal failure: the 'mature' rat model. Contrib Nephrol. 1988;60:46. 3345675 10.1159/000414789

[27] Niederhoffer N, Bobryshev YV, Lartaud-Idjouadiene I, et al. Aortic calcification produced by vitamin D3 plus nicotine. J Vasc Res. 1997;34:386–398. 9349732 10.1159/000159247

[28] Yokozawa T, Zheng PD, Oura H, et al. Animal model of adenine-induced chronic renal failure in rats. Nephron. 1986;44:230–234. 3785486 10.1159/000183992

[29] Quarles LD Role of FGF23 in vitamin D and phosphate metabolism: implications in chronic kidney disease. Exp Cell Res. 2012;318:1040–1048. 22421513 10.1016/j.yexcr.2012.02.027 PMC3336874

[30] Isakova T, Wahl P, Vargas GS, et al. Fibroblast growth factor 23 is elevated before parathyroid hormone and phosphate in chronic kidney disease. Kidney Int. 2011;79:1370–1378. 21389978 10.1038/ki.2011.47 PMC3134393

[31] Desjardins L, Liabeuf S, Renard C, et al. FGF23 is independently associated with vascular calcification but not bone mineral density in patients at various CKD stages. Osteoporos Int. 2012;23:2017–2025. 22109743 10.1007/s00198-011-1838-0

[32] Schoppet M, Hofbauer LC, Brinskelle-Schmal N, et al. Serum level of the phosphaturic factor FGF23 is associated with abdominal aortic calcification in men: the STRAMBO study. J Clin Endocrinol Metab. 2012;97:E575. 22319041 10.1210/jc.2011-2836

